# Characterizing Participant Perceptions about Smoking-Cessation Pharmacotherapy and E-Cigarettes from Facebook Smoking-Cessation Support Groups

**DOI:** 10.3390/ijerph19127314

**Published:** 2022-06-14

**Authors:** Allison Lee, Angela A. Chang, Joanne Chen Lyu, Pamela M. Ling, Stephanie L. Hsia

**Affiliations:** 1 School of Pharmacy, University of California, San Francisco, CA 94143, USA; allison.lee@ucsf.edu (A.L.); angela.chang@luhs.org (A.A.C.); 2 Center for Tobacco Control Research and Education, University of California, San Francisco, CA 94143, USA; chenjoanne.lyu@ucsf.edu (J.C.L.); pamela.ling@ucsf.edu (P.M.L.); 3 Division of General Internal Medicine, Department of Medicine, School of Medicine, University of California, San Francisco, CA 94143, USA

**Keywords:** tobacco-use cessation devices, e-cigarette, varenicline, bupropion, electronic nicotine-delivery systems, vaping, adolescent, social media

## Abstract

The prevalence of smoking among young adults aged 19–28 years old in the United States persists at rates of 14.3%. Young adults underutilize pharmacotherapy for smoking cessation, and the use of e-cigarettes has increased. We analyzed comments from online smoking-cessation support groups to understand young-adult smokers’ views of pharmacotherapy and e-cigarettes, to provide a more in-depth insight into the underutilization of pharmacotherapy. A qualitative analysis was performed on comments about pharmacotherapy and e-cigarettes from participants enrolled in online smoking-cessation support groups in 2016–2020. A codebook was developed with a deductive approach to code the comments, followed by thematic analysis. Eighteen themes were identified, with four dominant themes: interest, benefit, knowledge, and flavor. Participants expressed less interest in both nicotine-replacement therapy and e-cigarettes; moreover, they expressed unfamiliarity with and misconceptions about pharmacotherapy, and recognized the enticing flavors of e-cigarettes. Participants often felt e-cigarettes were not useful for smoking cessation, but the flavors of e-cigarettes were appealing for use. Participants had mixed opinions about the use of e-cigarettes for smoking cessation, but predominantly felt e-cigarettes were not useful for smoking cessation. The use of social media may be an effective way to address misconceptions about pharmacotherapy for smoking cessation and increase willingness to accept assistance.

## 1. Introduction

Though the prevalence of cigarette smoking among young adults has been decreasing over the past several years, as many as 14.2% of young adults age 19-28 in the United States (USA) still currently smoke cigarettes [1,2]. People who start smoking at a younger age are more likely to develop severe nicotine dependence later in life [3]. Early smoking cessation has shown positive health benefits, with a reduction in the risk of morbidity and mortality from comorbid conditions associated with tobacco smoking [4]. Currently, the gold standard for smoking cessation includes utilizing behavioral support in conjunction with pharmacotherapy [5,6,7]. Young adults have demonstrated more interest in or attempts to quit smoking compared to older adults, but young adults have lower success rates in smoking cessation as they are more likely to attempt to quit without assistance [1,8,9]. Hartmann-Boyce et al. conducted a systematic review that evaluated the efficacy of interventions for young adults to quit smoking and did not find strong evidence for any particular method of behavioral support or medication for long-term smoking cessation [10]. Studies have found that nicotine-replacement therapy (NRT)—including nicotine gum, lozenges, transdermal patch, nasal spray, and inhaler—and non-nicotinic pharmacologic therapies, such as bupropion and varenicline, are effective smoking-cessation aids; moreover, it is suggested that they increase the quit rate by 50–70%, regardless of participation in behavioral counseling [11]. However, pharmacotherapy has been underutilized in young adults when compared with older adults [1,10]. With regard to quit attempts, 7.6% of young adults reported using gold-standard smoking-cessation strategies, such as medications and behavioral counseling, compared to 23.3% of older adults utilizing such strategies [1]. There are currently limited qualitative studies exploring reasons for the lack of pharmacotherapy use in the young-adult population.

Watkins et al. found that young adults are 3.6 times more likely to use electronic cigarettes (e-cigarettes) or “vapes”, also known as electronic nicotine-delivery systems (ENDS), than evidence-based pharmacotherapy [1]. One possible explanation for the underutilization of smoking-cessation pharmacotherapy in young adults may be the perception that evidence-based medications are ineffective with undesirable side effects [12]. Another possible explanation for the underutilization of pharmacotherapy is that there is a preference for ENDS among young adults as a potential replacement for regular cigarettes, instead of pharmacotherapy [13,14]. ENDS are not FDA-approved for smoking cessation, but they have been promoted as smoking-cessation devices [15]. A few clinical trials have shown the efficacy of ENDS in smoking cessation. One study found ENDS to be more effective than NRT when used in conjunction with behavioral therapy [16]. A recent meta-analysis found that ENDS use was associated with smoking cessation in randomized clinical trials, but not in observational studies of their use in the population [17]. The Population Assessment of Tobacco and Health (PATH) Study found that individuals using ENDS had similar cigarette abstinence rates one to two years after quit attempt in comparison to individuals using NRT or no products [18]. The efficacy of ENDS for smoking cessation is still not well-established, and there are insufficient data surrounding the long-term health risks of the use of ENDS [19].

Behavioral support for smoking cessation has been found to increase an individual’s rate of smoking cessation from 10% to 20% [10]. Though face-to-face counseling has been reported as the most effective form of behavioral therapy, it is also the most underutilized service [20]. There has been a rise in the use of social-media platforms to engage users for health and wellness promotion, which has led to an increased interest in online smoking-cessation support groups, with early data showing promise for success in the young-adult population [21,22,23]. Social media can help promote mutual support and foster encouragement around a shared learning experience, while providing insight into individual perceptions around smoking-cessation methods. An evidence-based online smoking-cessation support group named *Commune Smokefree Social* was implemented utilizing the online social-media platform, Facebook. *Commune Smokefree Social* was an adaptation of the Tobacco Status Project, which had shown short-term efficacy in a randomized trial [24,25]. The content of participants’ comments in response to the programmed Facebook posts in the cessation groups provides a unique opportunity to study the perceptions of pharmacotherapy and ENDS among young adults utilizing online services to quit smoking. These insights may improve future smoking-cessation interventions by providing target areas for pharmacotherapy education, identifying needs for public-health education about ENDS, and subsequently improving the utilization of pharmacotherapy.

Specifically, the objective of this study was to characterize perceptions around NRT, non-nicotinic pharmacologic medications, and ENDS for smoking cessation among young adults participating in a social-media-based smoking-cessation intervention in the California Bay Area.

## 2. Materials and Methods

### 2.1. Participants

*Commune Smokefree Social* was made available for free to young adults (age 18–29) living in the San Francisco Bay Area (in the San Francisco, Alameda, Contra Costa, Marin, or San Mateo counties). Fifty-eight smoking-cessation support groups on Facebook with more than 800 young-adult participants took place between November 2016 and March 2020. The participants were assigned to either a “Getting Ready” or “Not Ready” group based on participants’ readiness to quit, as defined by willingness to make a quit attempt within 30 days of enrollment. Each Facebook group was active for a duration of three months. Participants who completed baseline and follow-up surveys received gift card incentives. Within the baseline survey, participants were asked one question from the Fägerstrom Test for Nicotine Dependence to generally categorize their nicotine dependence, “How soon after you wake up do you smoke your first cigarette?” All participants were encouraged to actively express opinions and engage in discussions in the groups. 

The *Commune Smokefree Social* Facebook groups were facilitated by trained smoking-cessation counselors. The intervention included daily Facebook posts for 90 days and weekly one-hour “live” sessions conducted by trained smoking-cessation specialists, which provided an opportunity to address participant questions in real time or provide tailored smoking-cessation advice. Starting in November 2019, weekly one-hour student pharmacist sessions were implemented for an additional opportunity to address questions regarding NRT and smoking-cessation medications. Post content was based on the U.S. Clinical Practice Guidelines for smoking cessation and the Transtheoretical Model of behavior change, and utilized images, videos, and text designed to reflect the experience of young-adult smokers and elicit participation [26,27]. The post content was tailored to readiness to quit, based on whether participants were “Getting Ready” or “Not Ready” to quit smoking. In the Getting Ready groups, posts focused on actionable items, such as setting a quit date or creating behavioral modifications; posts in the Not Ready groups were more encouraging and informational regarding the benefits of quitting smoking or the harms of smoking. In this study, we analyzed only posts from the Getting Ready groups based on the following considerations: (1) 59% of participants were placed in Getting Ready groups; (2) Getting Ready groups were more active and engaged in discussion of pharmacotherapy. Among the 377 participants in Getting Ready groups, 93% of participants completed the baseline survey. Table 1 displays the baseline survey demographics and smoking characteristics of the participants, including all quit methods ever attempted. The University of California San Francisco Institutional Review Board (IRB) reviewed this program and determined that IRB approval and informed consent were not necessary.

### 2.2. Data Collection and Analysis

This was an analysis of qualitative data collected as part of quality improvement efforts for the clinical smoking-cessation service. Comments were extracted from the 377 participants in the Getting Ready groups. These were obtained from each group’s web browser code using “Developer Tools” in Google Chrome. Comments were then extracted using MATLAB^®^, a data analysis platform, and compiled into an Excel spreadsheet. Comments were successfully extracted from 33 Getting Ready groups. Two authors read the full sets of posts and comments for each group, identified comments about NRT, smoking-cessation medications, and e-cigarettes, and saved them in a separate transcript document. To ensure complete data extraction, search terms including “e-cigarette”, “vaping”, “patch”, “gum”, “lozenge”, “inhaler”, “nasal spray”, “bupropion”, “varenicline”, “Wellbutrin”, and “Chantix” were utilized to identify relevant comments in the Excel spreadsheets. Qualitative analysis was conducted in Dedoose version 8.3.45 (SocioCultural Research Consultants, Los Angeles, CA, USA). 

We took a deductive approach and developed a code book based on review of the scientific literature and an initial review of four transcript documents. Codes and definitions were reviewed and discussed iteratively by the research team, and the code book was revised (Table 2). Each document was then coded by two independent coders identifying common themes. Weekly meetings were held among the research team to discuss any differences in coding or interpretation of the themes. The coders then pilot-tested the theme list in another four documents to ensure it was exhaustive. After iteratively coding texts and resolving discrepancies together with a third reviewer, a final codebook of themes was generated and entered, and subsequently used to code all transcripts. The first 20% of the Facebook comments were double coded by both researchers; discrepancies were reviewed and resolved, and, if necessary, coding differences were resolved by discussion with a third reviewer. Coding and discussions were repeated iteratively with a final interrater reliability of 85%. The final codebook was then applied to the rest of the data [28,29]. A thematic analysis was performed on the coded data utilizing Dedoose’s code co-occurrence tools.

## 3. Results

There were a total of 427 total posts focused on NRT and ENDS within the 33 groups. Eighteen common themes were identified across all groups (Table 2), and there were four most-prevalent themes identified: (1) interest, (2) benefit, (3) knowledge, and (4) flavor. In addition, we identified common co-occurrences between knowledge and NRT and smoking-cessation medications, as well as a co-occurrence of the theme of flavor with excerpts focused on ENDS (Table 3). Participants generally expressed disinterest in the utilization of NRT unless they had a history of NRT use and found it to be helpful with quitting. There was a general perceived disinterest and lack of usefulness in ENDS. Participants also showed a lack of knowledge regarding certain NRT formulations, as well as misconceptions about NRT use. Lastly, the flavors of ENDS were found to be appealing for use.

### 3.1. Interest

Participants demonstrated little interest in using some NRT and ENDS. Most participants expressed disinterest towards the use of bupropion, varenicline, and a nicotine nasal spray when compared to nicotine patches, gum, or lozenges. Participants reported that using nasal sprays in general was an unappealing option, and expressed that they typically avoid this form of drug administration, even when treating the common cold or allergies. One participant stated:

*“don’t really like the idea of spraying nicotine in my nasal cavity.”*

There were concerns regarding side effects and cost of NRT or medications, which deterred participants from starting or continuing pharmacotherapy. Side effects that were commonly reported included skin irritation from nicotine patches, and mouth irritation and nausea from nicotine lozenges and gum. Participants who expressed disinterest in NRT tended to express a preference for quitting cold-turkey (i.e., without assistance from any aids). Other individuals expressed disinterest in utilizing pills for smoking cessation, and believed they could quit on their own through habit changes alone. Participants often felt that there was no need for NRT because they did not think they were chemically dependent on nicotine.

*“I’ve never heard of it [bupropion], but since nicotine dependence isn’t my main issue with quitting, I don’t know that it would be right for me.”*

Despite more participants expressing the higher frequency of disinterest in NRT, there were some participants that expressed a willingness to consider using NRT, with the highest interest being in nicotine lozenges.

*“Haven’t tried any over the counter method but it [lozenge] does seem effective because you really just miss the feeling of a cigarette in your mouth too.”*

Posts about ENDS revealed the concern about the safety and health risks of ENDS use, which contributed to overall disinterest. Conversations about ENDS differed from those regarding NRT, in that participants were particularly focused on safety when discussing ENDS. Several individuals shared their skepticism about joining the ENDS trend and concerns over the potential occurrence of “popcorn lung” (also known as bronchiolitis obliterans, which is a scarring and narrowing of the air sacs in the lungs typically resulting from chemical exposure). Comments about ENDS and NRT were similar in that many individuals were less interested because of doubts regarding efficacy for quitting. In general, more participants expressed disinterest towards ENDS than interest. Individuals who expressed interest shared that ENDS were an appealing alternative compared to more foul-smelling cigarettes. There was an association between a sense of shame with smoking cigarettes and the general notion that ENDS were a more socially acceptable way to mask a nicotine addiction.

*“I’d probably grab the e-cig. It’s easier to hide and some of the flavors are good.”*

### 3.2. Benefit of Pharmacotherapy for Cessation

Participants who had personal experience with NRT, bupropion, or varenicline tended to report beneficial outcomes with their use compared to participants with no experience. Participants expressed mixed impressions of nicotine patches and nicotine gum; many expressed that these forms of pharmacotherapy were useful, while others expressed that they were not beneficial. Individuals found the nicotine patch and gum to be helpful in preventing nicotine cravings. Those who stated that nicotine gum was useful had personal experience with utilizing gum to help them quit, particularly to help satisfy the hand–mouth action, preventing further cravings for more cigarettes.

*“I like gum because it also satisfies my mouth’s need to be active without shoving a bunch of food into it.”*

Some participants stated that the nicotine patch was not effective in keeping their hands distracted from reaching another cigarette. Individuals also expressed that nicotine patches were not effective often, because their use was limited by the side effects and inability to keep patches on for a longer duration. Participants who stated that nicotine gum was not useful often expressed an aversion to the peppery taste.

*“I did but it [nicotine patch] made me sick and gave me a rash that would last for weeks each time I used one. I’ve known it [nicotine patch] to work for other people though.”*

The attitudes and perceptions around ENDS shared many similarities with those around NRT. There was a strong correlation with friend and family influences that led participants to believe ENDS were an ineffective aid to quit smoking cigarettes. Participants also mentioned that ENDS helped curb their craving for a cigarette, similar to their thoughts about certain NRTs. Other individuals expressed that ENDS were ineffective as an alternative due to the throat burn and overall feeling of being unsatisfied. In contrast, many participants mentioned that they tried ENDS, but expressed concerns over them perpetuating the dependence due to their similarity to cigarettes.

*“E-cigs don’t work for me, they’re too similar and its [sic] too easy to spend all day vaping.”*

### 3.3. Knowledge

Participants expressed more familiarity with nicotine patches and gums and less familiarity with other forms of NRT, such as nicotine inhalers and nasal sprays. Many of those who lacked knowledge about NRT and medications expressed less interest in utilizing these options to aid in smoking cessation. There were many misconceptions about NRT or medication use, such as the belief that NRT would cause more dependence on nicotine or cause a new dependence. Some participants expressed distaste for the utilization of smoking-cessation medications because they viewed it as a replacement rather than an aid to quit smoking. There was a common belief that nicotine is a harmful substance and that utilizing NRT would be counterintuitive to quitting smoking.

*“Not interested in replacing the intake method... I don’t want nicotine in any form to rule my life, and I am afraid I will simply just use the patch or the gum or the lozenge indefinitely.”*

The co-occurrence of knowledge and cost appeared, which suggested that participants were unaware of options that would help NRT and medications become more affordable, such as insurance coverage. Many individuals did not know that certain smoking-cessation pharmacotherapy options are covered under insurance plans.

*“Just didn’t like the fact it [nicotine gum] was $60 per box and I didn’t know it [nicotine gum] was covered by insurance at the time.”*

In contrast, there was an even mixture of participants who were aware of ENDS and those who were unaware of this nicotine-delivery system. Many individuals who knew about ENDS were those who had prior experience with them. 

### 3.4. Flavor of ENDS

Participants believed that the use of candy or fruit flavors was a smart marketing strategy and were enticing, and expressed that the ENDS flavors made their smoking experience more palatable and enjoyable than smoking cigarettes. Many shared that their utilization of ENDS was not for purposes of smoking cessation and felt that ENDS were harmless.

*“I enjoy the flavors and love how not stinky it is compared to cigs. I hope to gradually reduce my use but I enjoy it.”*

However, there were a few participants who expressed disgust towards the flavors used in the ENDS juices and their potential for harm.

## 4. Discussion

To our knowledge, this is the only qualitative analysis, to date, that describes perceptions surrounding smoking-cessation pharmacotherapy and ENDS in young adults attempting to quit smoking on social media. Our data analysis identified gaps in knowledge, which were mainly misconceptions about NRT and smoking-cessation medications. Our findings were similar to Duarte et al.’s focus-group study with non-college-educated young adults, which revealed an overall negative perception about cessation treatments due to side effects, and a misunderstanding of how these pharmacologic therapies work [30]. Our analysis identified particular forms of NRT that coincided with the negative experiences in young-adult participants. Side effects with nicotine patches, including skin irritation and nightmares, were shown to limit participants’ continued use or interest. Additionally, there were frequent posts regarding nicotine gum and its taste, often described as “peppery.” This may be related to the improper use of nicotine gum, as the recommended method is “park and chew” to limit the “peppery” taste [31]. Though there has been discussion on the international front regarding the withdrawal of varenicline from the market due to nitrosamine detection, participants did not note this concern within the Facebook groups. Moreover, in the United States, varenicline is still widely used, as the FDA has deemed current nitrosamine levels as acceptable and safe [32]. Brunette et al. characterized attitudes about smoking-cessation pharmacotherapy among young-adult smokers with severe mental illness, and found that through motivational and educational interventions, participant perceptions of pharmacotherapy improved. However, the study subjects still attempted to quit without pharmacologic therapy [33]. Understanding the barriers in these young-adult smokers to utilizing pharmacotherapy suggests it is crucial to provide more effective education, to mitigate misconceptions and educate young adults on how to properly use NRT. 

Participants expressed interest in ENDS as an alternative to tobacco cigarettes because of the enticing and customizable flavors of ENDS. This was consistent with a survey conducted by Farsalinos et al., which found that the most important factor for the maintenance of ENDS use was the variety of flavorings, which assisted with reduction of tobacco cigarette use [34]. However, most participants perceived ENDS as not beneficial because participants were more likely to try to decrease all nicotine products as part of their quit attempts. This is consistent with findings from a focus-group study that characterized perceptions about ENDS, which found that young-adult smokers were aware of both the positive and negative qualities of vaping [35]. Several participants in our study also expressed skepticism about ENDS’ safety and efficacy for smoking cessation. However, the posts in the Facebook groups may have contributed to this perception of ENDS, as many of the programmed posts discussed the negative side effects and efficacy of ENDS. The National Academies of Sciences, Engineering, and Medicine found that ENDS had fewer short-term adverse health effects compared to tobacco cigarettes, but the overall long-term adverse health effects remain unclear [36]. With the increasing interest in ENDS as a smoking-cessation method, clinicians will need to be prepared to discuss the risks, including the uncertainty of long-term safety effects, and potential benefits to assist with complete cessation of tobacco cigarettes [7,37].

In addition, non-pharmacological methods may help decrease young-adult smoking, including motivational cognitive behavioral therapy or social-media campaigns that de-normalize the tobacco industry [38,39,40,41]. Due to the underutilization of pharmacotherapy paired with behavioral modifications in the young smoker population, educational campaigns to address misconceptions about NRT and smoking-cessation medications may be useful. This study identified barriers and concerns with pharmacotherapy use in young adults that could inform future social-media interventions, such as addressing nicotine dependence and providing medication education for correct and effective pharmacotherapy use. In a recent systematic review, social-media-based smoking-cessation approaches were found to be effective, with a higher tobacco abstinence rate after intervention [42]. Since social media are the favorite media among young adults, educational campaigns about ENDS and smoking-cessation pharmacotherapies targeting this population should be considered. It may be beneficial to further explore various platforms with higher popularity, such as TikTok or Instagram. In addition, further research is warranted to address the safety and efficacy of ENDS for smoking cessation among young adults.

This qualitative analysis of Facebook posts is subject to inherent limitations. The themes identified were based on the content of the posts, and the coders may have brought biases in determining the themes; however, this was mitigated by blinded coding and having a third reviewer. Additionally, this intervention was limited to the young-adult population in the Bay Area. We cannot generalize our findings to other populations. Lastly, engagement among participants varied, so it is possible that the common themes that we identified may have been based on the more active participants. 

## 5. Conclusions

Many young-adult smokers ready to quit in online support groups expressed a lack of interest and knowledge in and about smoking-cessation pharmacotherapy. This was mainly driven by misconceptions about its use, side-effect concerns, a preference to quit without assistance (“cold turkey”), and a lack of recognition of nicotine dependence. In general, there were mixed opinions about ENDS, but more participants ready to quit expressed that ENDS would not be beneficial in smoking cessation. 

Social-media-based approaches for smoking cessation have been found to be an effective intervention, and their use may serve as a vehicle to deliver educational information regarding smoking-cessation pharmacotherapy to young adults. It is pertinent to identify effective methods to decrease smoking rates in young adults through online support groups. This may be achieved by integrating information for available smoking-cessation strategies to encourage tobacco abstinence, to prevent further risks of tobacco-related conditions and mortality. Social-media-based approaches should also address the barriers or misconceptions about pharmacologic agents that have been identified in our study and other literature, which may be significant barriers to young adults quitting smoking. 

As smoking among young adults is a major public-health issue across the world, efforts to use social media to mitigate incorrect, pre-perceived notions about smoking-cessation pharmacotherapy, and to promote the effectiveness of evidence-based smoking-cessation pharmacotherapy methods, are needed. 

## Figures and Tables

**Table 1:** Characteristics of Ready to Quit Participants Completing the Baseline Survey.

Characteristic	Participants (n = 349)
Male, n (%)	140 (40.1)
Age (years), mean (SD)	26 (5)
Race *, n (%)	
White	193 (55.3)
Hispanic	57 (16.3)
Black	61 (17.5)
Pacific Islander/Hawaiian Native	15 (4.3)
American Indian/Alaskan Native	21 (6.0)
Asian	60 (17.2)
Other	30 (8.6)
Annual household income, n (%)	
≤$40,000	204 (58.5)
$41,000–$80,000	80 (22.9)
$81,000–$200,000	48 (13.8)
>$200,000	9 (2.6)
Not reported	8 (2.2)
Education, n (%)	
High school or less	69 (19.8)
College	231 (66.2)
Higher than college	46 (13.2)
Not reported	3 (0.8)
Smoking characteristics	
Daily smoker, n (%)	159 (45.6)
Daily cigarette consumption, mean (SD)	3.6 (5)
Time to first cigarette, n (%)	
After 60 min	196 (56.2)
31–60 min	50 (14.3)
6–30 min	60 (17.2)
Within 5 min	43 (12.3)
Quit methods ever tried n (%)	
Nicotine gum	90 (25.6)
Nicotine patch	77 (22.1)
icotine spray	3 (0.9)
Nicotine inhaler	3 (0.9)
Bupropion/Zyban/Wellbutrin	27 (7.7)
Varenicline/Chantix	7 (2)
E-cigarette (with nicotine)	133 (38.1)
E-cigarette (without nicotine)	54 (15.5)
Hypnosis	10 (2.9)
Acupuncture	17 (4.9)
“Cold turkey”(abrupt cessation without assistance)	295 (84.5)
Gradually cut down	229 (65.6)
Stop smoking class/program for a fee	7 (2)
Stop smoking class/program for no fee	23 (6.6)
Advice or counseling from a physician, nurse, psychologist or other health professional	43 (12.3)
Telephone hotline	14 (4)
Online program	7 (2)
Texting program	5 (1.4)
Phone application	35 (10)
Other	14 (4)

* Participants were asked to indicate all that apply to them.

**Table 2:** Themes identified in the *Commune Smokefree Social* Facebook groups regarding smoking-cessation pharmacotherapy and e-cigarettes.

Theme	Description
Interest in NRT/Smoking-Cessation Medication	Interest or disinterest in use of NRT and smoking-cessation medications
NRT/Smoking-Cessation Medication Benefits	Useful/helpful or not useful/not helpful with NRT and smoking-cessation medications use to help quit smoking
Knowledgeable about NRT/Smoking	Level of knowledge about NRT and/or smoking-cessation medications
Cessation Medications	Nicotine patch, nicotine lozenge, nicotine gum, nicotine inhaler, nicotine nasal spray, bupropion or Wellbutrin, varenicline or Chantix
E-cigarette Interest	Interest or disinterest in use of e-cigarettes
E-cigarette Benefits	Usefulness/helpfulness or lack of usefulness/helpfulness of e-cigarettes to help quit smoking
Knowledgeable about E-cigarettes	Level of knowledge about e-cigarettes.
Side Effects	Adverse effect experienced from NRT, smoking-cessation medications, or e-cigarettes
Taste	Opinions on the taste of NRT, smoking-cessation medications, or e-cigarettes
Family/Friend Influence	Level of interest or disinterest in use of NRT, smoking-cessation medications or e-cigarettes based on hearsay from family member’s and/or friend’s personal experiences
Cost	Mention of the cost of NRT, smoking-cessation medications or e-cigarettes
Ambiguity	Uncertainty of interest or potential benefits in the use of NRT, smoking-cessation medications or e-cigarettes to assist with smoking cessation
Smell	Smell of e-cigarettes or lack of smell with smoking-cessation medications and NRT
Cold Turkey	Abrupt smoking-cessation without any assistance from the use of NRT, smoking-cessation medications or e-cigarettes as aids
Teachable Moments	Dialogue between participants and facilitators sharing new knowledge about NRT and smoking-cessation pharmacotherapy
Conditional/Last Choice	Consideration of the use of NRT or smoking-cessation medications if all other resources were exhausted
Flavor	Opinions on flavor of NRT or e-cigarettes
Nicotine Dependence	How dependent on nicotine participants perceive themselves to be

**Table 3:** Representative quotations displaying prevalent themes identified in *Commune Smokefree Social* transcripts.

Themes	Co-Occurring Themes	Quotations
Interest	Cost, side effects, cold turkey, not nicotine dependent	*“Nope, both have horrible side effects, but they spin it off as no big deal, cancer free ciggs [sic] no cool, blood bubbles in your lungs and more likely the device blows up in your face, not safe no cool”*
Benefit	Friend/family influence, side effects	*“This pill actually really worked for me when I took it. Its brand name is Chantix. For me, it curbed the reward of smoking, since it works by blocking nicotinic receptors in our brains. When I smoked on chantix, it tasted unbearable and I actively didn’t want to take additional drags. I’d recommend it to anyone trying to quit. I think it’s also covered by most health insurance formularies bc [sic] it’s a preventative solution to [sic]”* *“I’ve used a high dosage of nicotine patch before, beyond the regular intake from smokes. It did not work for me over two weeks and I felt compelled to smoke, even though my nicotine intake was higher than normal.”*
Knowledge	Misconception, cost, not nicotine dependent	*“I’ve honestly not heard much about nicotine inhalers, unless vapes fall into that category. I’d imagine they work similarly, without the exhalation of smoke vapor.”* *“But sounds gross. It’s getting easter [sic] to just not smoke than find a replacement”*
Flavor	Taste	*“They’re gross! I don’t like flavored tabacco [sic]”*

## Data Availability

The data presented in this study are available on request from the corresponding author. The data are not publicly available due to privacy reasons.
